# Applying the Intersectionality Lens to Understand Minority Ethnic Women's Experiences of the Breast Cancer Care Pathway in England: A Qualitative Interview Study

**DOI:** 10.1002/pon.70092

**Published:** 2025-02-06

**Authors:** Mar Estupiñán Fdez. de Mesa, Maureen Ferguson, Saran Green, Afrodita Marcu, Emma Ream, Katriina L. Whitaker

**Affiliations:** ^1^ School of Health Sciences University of Surrey Surrey UK; ^2^ PPI Representative London UK; ^3^ Patient Representative King's College London London UK

**Keywords:** Black Feminist Thought, breast cancer, cancer care, health equity gap, inequalities, intersectionality, minority ethnic women

## Abstract

**Background:**

Exploring the role of structural power in relation to an individual's location in society can yield novel insights into cancer inequities. We aimed to understand how minority ethnic women's identities (age, gender, ethnicity, social position) intersected with social networks and healthcare services to influence their experiences of breast cancer care.

**Methods:**

Semi‐structured interviews with 20 women aged 31–60 years with a breast cancer diagnosis identifying as: Asian (*n* = 7), Black (*n* = 9), and of mixed ethnicity (*n* = 4) about their breast cancer journey. Data were analysed using Framework Analysis.

**Results:**

We developed four themes; feeling stereotyped by (a) healthcare professionals (HCPs) and (b) social networks; influence of HCPs' knowledge, attitudes, and behaviours; perceived barriers in healthcare services; and influence of cultural beliefs. We provided a visual representation to illustrate the multifaceted factors that explain pathways to breast cancer inequities for these groups.

**Conclusion:**

Our findings underscored that minority ethnic women negotiated complex processes that influence their coping responses, access to services, and management of their disease. Our study exposed institutional deficiencies that systematically disadvantage minority ethnic women. These findings suggest that policymakers and clinicians should reformulate policies and cancer services to ensure all women with breast cancer receive equal and optimal care.

## 1 Introduction

In high‐income countries with comprehensive and high‐quality healthcare systems, whether publicly or privately funded, breast cancer inequities exist [1]. These are unfair and avoidable health differences among groups arising from the influence of social factors [2]. Much of the research to examine these inequities has centred on prevention and screening [3], treatment [4], tumour characteristics [5], and survival [6]. However, to date insufficient attention has been paid to the role that sociohistorical processes and power relations may play in shaping the lived experience of minority ethnic women along the continuum of care [7]. Furthermore, the understanding of inequities in breast cancer care and outcomes is largely based on the norms of the dominant White ethnic group [8]. That is, using a comparative approach that conceives whiteness as the norm and defines minority ethnic women as they stand in relation to that group.

In recent years, cancer in women has become a global focal point in broader conversations surrounding the insufficient regard it has received compared to other diseases affecting women's health [9]. There is an increasing recognition that multiple forms of discrimination combine in myriad ways to influence opportunities to seek and receive prompt diagnosis and quality cancer care for women [7]. For instance, evidence suggests that minority ethnic women are more likely to experience inequities in cancer care due to the double jeopardy effect [10]. That is, the compounded discrimination (e.g., racism *and* heterosexism) individuals face by virtue of belonging to multiple marginalised groups (e.g., ethnicity/sexual orientation) [11].

Similarly, discussions to challenge the status quo of breast cancer inequities and a call for action to improve the care and outcomes of minority ethnic women are gaining considerable traction [9, 12]. To inform these conversations, there is a need to understand the multifaceted aspects of minority ethnic women's identity and how these factors interlock with socio‐institutional practices to shape unique experiences of breast cancer inequity. Only if we shift our research focus to specifically ask minority ethnic women to share their views and perceptions on the care they receive, we will identify potential deficiencies in the healthcare system and the role of social norms and culture on living with breast cancer [7].

Black Feminism Thought (BFT) and intersectionality are two frameworks that facilitate this shift in research. The BFT emerged as a movement to create space for women of colour to, individually and collectively, make sense of and legitimise their historically discounted experiences [13]. The theory provides a sociohistorical lens to understand the experiences of minority ethnic women and how they negotiate their intersections of age, gender, ethnicity, and social position in the healthcare context [14]. On the other hand, intersectionality is one theoretical framework that has been fundamental to conceptualise how multiple systems of discrimination interconnect to disadvantage some populations more than others in society [15]. Furthermore, intersectionality allows researchers to go beyond individual risks to explore the ways in which individual identities compound with the social determinants of health and institutional services to influence breast cancer care and outcomes [16].

To contribute to the literature, we aimed to understand how individual identities (age, gender, ethnicity, and social position) intersected with structural power (social networks, cancer services, and clinical practice) to shape unique experience of cancer inequity for minority ethnic women receiving care in a publicly funded health system in England. Our research is context specific; however, England shares commonalities with other high‐income countries. Evidence suggests that power hierarchies and discriminatory systems related to health inequities are similar across contexts, countries, and history [17]. Therefore, we are confident that our findings will be valuable for policymakers, clinicians, and researchers beyond England.

## 2 Methods

### 2.1 Study Design

Semi‐structured interviews, to facilitate exploring the complex and nuanced lived experience of minoritised groups. Digitally recorded interviews were conducted online by the first author and lasted on average 53 min (35 min to 1 h 08 min). A protocol developed in line with previous research on minority ethnic women [18] guided the interviews (Table S1). Questions were designed to minimise the risk of deconstructing participants' identity (e.g. asking about participants' experience as a Black women, instead of the experience as being Black and, separately, as being a woman) [19]. To avoid further stigmatising participants during the interview process [20], the topic guide was discussed with Black and Asian stakeholders. This process assisted tailoring the tone and language. The topic guide was divided in two sections: background and the breast cancer pathway.

### 2.2 Sample and Recruitment

We recruited women (≥ 18 years) who self‐identified as of Black and Asian backgrounds (including mixed ethnicities) and lived in England; henceforth, referred to as minority ethnic women unless stated otherwise. Women were eligible if they had received a breast cancer diagnosis within the previous 10 years and had used the publicly funded breast cancer service or a combination of public and private cancer services. Participants were excluded if they had not used the public cancer services, and/or did not speak English.

Between September 2023 and May 2024, participants were recruited through purposive sampling and snowballing to obtain a diverse sample and representative spread of age, ethnicity, social position (proxy dimension—education), and geography. Recruitment was via professional and charity networks, patient representative groups, and online advertising. Women completed a short online questionnaire to express interest in participating and provided information which was used by the first author to screen women for eligibility. Women were presented with a participant information leaflet and consent form providing details of the study. All women gave verbal consent at the start of the interview and were compensated with a £25 gift voucher for their time.

### 2.3 Data Analysis

Transcribed interviews were managed using NVIVO qualitative software (QSR v.12). The interviews were transcribed verbatim, anonymised, and analysed using Framework Analysis [21] which entails (1): familiarising with the data (2), identifying a thematic framework (3), indexing (4), charting and (5) mapping and interpreting the data. The first author inductively coded the transcripts line‐by‐line and as the data were collected. Subsequently, informed by underpinning theories guiding the study, a framework was developed using axial and pattern coding [22] and comprised the main themes and categories. Attention was given to concepts such as power, social context, lived experiences that explicitly or implicitly influenced unique outcomes based on participants' social locations, and how participants' concerns related to systems of oppression [20, 23]. Analysis included vertical and horizontal comparison to identify similarities and differences between and within participants (e.g., comparing the experience between participants younger than 50 years [ineligible for breast screening] and, separately, younger compared to 50 years and older [eligible for breast screening]). The research team reviewed the data to assess the themes and categories aligned with the participants' quotes. In discussion with the research team, disagreements were resolved by consensus. Field notes supported the interpretation and contextualisation of the data. Quotes were used to illustrate the findings, which were minimally edited for readability. Participants were allocated unique identifiers (ID number), and these are presented next to each quote. We followed the Standards for Reporting Qualitative Research Checklist (SRQR)—that is, an evidence‐based checklist consisting of 21 items [24] (Table S2).

### 2.4 Positionality and Reflexivity

The research team comprised four female academics with diverse backgrounds (public health, psychology, and nursing). The first author is a White Other, middle‐aged, immigrant, and non‐native English speaker. Despite the lack of shared cultural background and experience of breast cancer, the first author (the interviewer) was able to elicit trust and rich narratives from the participants, and thus contributed to the joint construction of meaning [18]. However, participants may have still omitted some details, or the researcher may have misinterpreted participants' meaning. Therefore, working with the community (see next section), including two representatives co‐authoring this article, was instrumental to address these challenges.

### 2.5 Patient and Public Involvement (PPI)

The first author's longstanding relationship and established collaboration with the community helped build rapport and trust with participants. They met and consulted a breast cancer survivor of Black Caribbean heritage and two PPI groups throughout the research process. These PPI representatives provided feedback on the topic guide (e.g., framing questions), reviewed and provided input on public‐facing documents (e.g. co‐producing the recruitment poster to make it culturally appropriate), assisted with the recruitment process, and validated the interpretation of findings.

## 3 Results

### 3.1 Characteristics of Participants

Table 1 shows the sample characteristics. We recruited 20 participants with a self‐reported breast cancer diagnosis. Age ranged from 31 to 60 years (mean age = 48.7). The majority were UK‐born second‐generation (*N* = 13, 65%) and resided in various geographical areas of England (55% in London). There was an approximately even representation across age (5% ≤ 34 years; 35% 35–44 years, 30% 45–54 years, and 30% ≥ 55 years), ethnicities (45% Black, 35% Asian, 20% mixed ethnicities), and educational attainment (30% school/college education level (SC); 30% undergraduate degrees (UG); 40% postgraduate degrees (PG)). Routes to diagnosis varied and most had solely used publicly funded services. In addition, participants were at different stages of their cancer journeys, and most had received a diagnosis ≤ 5 years.

**TABLE 1:** Characteristics of participants.

Sample characteristics	Total	%
Age (mean age = 47.5; range from 31 to 60 years)		
16–34	1	5
35–44	7	35
45–54	6	30
≥ 55	6	30
Ethnicity		
Asian	7	35
Black	9	45
Mixed ethnicities	4	20
Ethnicity—Subgroups		
Indian	3	15
Pakistani	1	5
Bangladeshi	1	5
Any other Asian background	2	10
Caribbean	4	20
African	3	15
Any other Black, Black British, or Caribbean background	2	10
White and Black Caribbean	1	5
White and Black African	2	11
Any other mixed or multiple ethnic background	1	5
Generation		
First generation	6	30
Second generation	13	65
Third generation	1	5
Sexual orientation		
Heterosexual or straight	17	85
Bisexual	1	5
Not given/not known	2	10
Education		
School & college	6	30
Undergraduate degree	6	30
Postgraduate degree	8	40
Geographical area		
London	11	55
South East	4	20
North West	1	5
West Midlands	2	10
East England	2	10
Route into diagnosis		
Self‐referral	11	55
Screening programme	5	25
Incidental (visit HCPs for other reasons)	3	15
Private service (routine mammogram)	1	5
Time since diagnosis		
≤ 1 year	7	35
2–5 years	11	55
6–10 years	2	10
Breast cancer services		
NHS breast cancer services	17	85
NHS breast cancer services and private services	3	15

### 3.2 Themes

Most participants had an overall positive view of the healthcare system. However, participants shared many instances of negative experiences along the continuum of care, including feeling of being treated differently due to their intersecting identities relative to gender, ethnicity, and social position. We also identified other factors relating to participants' networks (family, friends, and community) which distinctively shaped their experiences of diagnosis and management of their disease. We present four major themes including: feeling stereotyped by (a) healthcare professionals (HCPs) and (b) social networks; influence of HPCs' knowledge, attitudes, and behaviours; perceived barriers in healthcare services; and influence of cultural beliefs. In Table 2, we provide additional quotes per theme.

**TABLE 2:** Themes and representative quotes.

Theme	Sub‐theme	Quote
Feeling stereotyped by healthcare professionals (HCPs) and social networks	Autonomy and competence	*‘“We're medical, we know this is what you need”… And then one of the oncologists questioned whether I had capacity to make decisions and I was really offended by that’. (ID19; 53 years, Mixed ethnicity, UG)*
	Differences in communication styles	*‘The fact that you might make somebody feel like you're questioning their profession and that isn't the case…then you're marked in a certain way. “Agh, it's that woman coming in again?” “Yes, it's me”’. (ID07; 50 years, Other Black, PG)*
	Strong Black woman	*‘I think they [HCPs] perceive Black women as these strong and all knowing, capable, if anything, women, when actually that is not. I mean, yes, we are strong women, we have to be, but that is not always the case. I don't wanna always be strong and sometimes I'd like someone advocates for me or stands up for me or looks out for me’. (ID20; 57 years, Black Caribbean; UG)*
Experiences with cancer services	Insufficient medical knowledge	*‘I think they [HCPs] should be taught actually how to understand people of colour's needs and understand that they're different to English people, uhm…I think that should be established in their training. That they can better understand us and treat us appropriately’. (ID20, 57 years, Black Caribbean, UG)*
	Medical silencing	*‘She [consultant] was dismissive… “We call you in two years,” I said, “well, I wouldn't be very happy about that.” Very dismissive and I have experienced a lot of racism in my life, and it really felt me being a Black woman, I didn't count’. (ID02; 60 years, Mixed ethnicity, PG)*
	Microaggressions, interpersonal racism	*‘She [nurse] showed me a white one [prosthesis]… we're talking about your breast as being removed and this is what we've got for you…They would never have shown a White person a Black breast prosthesis’. (ID04; 38 years, Black African, UG)*
Perceived barriers in healthcare services	Primary care	*‘I felt with the GP…I definitely knew that lump was nothing to do with my period, and I definitely felt that she didn't really understand…“so you have to come back”. And so there was still that delay because…I had to see her more than once for her to refer me’. (ID03; 37 years, Asian Indian, PG)*
	Lack of tailored services	*‘Before I had the reconstruction, they give you the prosthesis, the skin tones are always for White counterparts. It doesn't match our skin. It's very trivial. It's small, but when you're going through that big body change, it does matter…’. (ID10; 59 years, Asian Indian, UG)*
	Workforce diversity	*‘If you have an ethnic background and you go into a hospital, you don't see many consultants of my colour at the forefront, right? So, you'll then walk in… I can't steer away from that. I think you just don't see people of our colour at the senior level and, and that's some of the problem as well’. (ID07; 50 years, Black other, PG)*
Influence of cultural beliefs	Taboo and hidden condition	*‘[Participant's mother] “No, we don't tell anybody, and nobody needs to know” …I was really angry…and then, my partner, he is a Black Caribbean, and although I didn't have the same reaction, it was similar in that “We don't talk about it” …it's that cultural understanding, embedded culture that we don't talk about cancer. It's such a hidden thing…’. (ID13; 41 years, Asian, UG)*

Abbreviations: PG, postgraduate degree; SC, school & college; UG, undergraduate degree.

#### 3.2.1 Feeling Stereotyped

Participants perceived they were stereotyped within the healthcare system and to a certain extent within their own networks. Factors that explained this feeling included HCPs' assumptions on the one hand, and cultural pressures on the other. Participants perceived these assumptions, and cultural pressures were linked to their social locations (based on the intersection of gender, ethnicity, and social position).

##### 3.2.1.1 HCPs

Despite participants' agency to self‐advocate during medical consultations, they felt that HCPs' assumptions about minority ethnic women questioned their sense of autonomy and competence.

She [consultant] wasn't focusing on me as a patient…she said… ‘you're a student or carer, aren't you?’ …it was almost like she was just lumping me into this thing of stereotypes…, you know, Black woman must be a carer…that hurt me.

(ID05; 45 years, Black African, PG)

Other participants perceived that interpersonal relationships could be affected due to differences in communication styles between patients from different ethnic backgrounds and HCPs. In some instances, participants explained they opted for self‐silencing to avoid being misunderstood. The opposite was also true; that is, when participants interacted with professionals from the same ethnic background, they felt better comprehended, even when using non‐verbal behaviour.

Sometimes when we speak to a White professional, kind of hold back because…they might take offense to how you're saying it, or they may not understand…I had another appointment and [the nurse] was Black…I just felt like, you know what, you just exhale…That's how I felt with [the Black nurse], but I didn't realise I was holding my breath when I was with [the White nurse].

(ID16; 40 years, Black Caribbean, SC)

##### 3.2.1.2 Social Networks

Many participants experienced emotional toll due to societal expectations that women from their ethnic group should be strong (i.e., ‘Strong Black Women’) to cope with breast cancer. This cultural stereotype was largely experienced by women from Black and mixed ethnicity backgrounds:

This notion of Black women being strong, the amount of people that said ‘You're strong, you're gonna be OK’ really annoys me. Because that's the denial of self. Why do I have to be strong?…I've got cancer. I'm fragile, I don't have to be strong. I just need to gain the support that I need…

(ID02; 60 years, Mixed ethnicity, PG)

#### 3.2.2 Influence of HCPs' Knowledge, Attitudes, and Behaviours

A common theme discussed by participants was the insufficient medical knowledge on the part of HCPs relating to how breast cancer manifests on minority ethnic women and the lack of ethnic‐specific information to manage the disease in these groups. This situation created some confrontational experiences between participants and HCPs and made participants question the equity in service provision.

The peau d'orange I could see it…and he [surgeon] didn't pick up on the colour or the peau d'orange…He wasn't worried, he didn't think it was anything…and I didn't feel that the surgeon gave me the opportunity to point out additional symptoms.

(ID04; 38 years, Black African, UG)

In other instances, participants felt HCPs did not listen or dismissed their concerns (i.e., medical silencing [25]), or objectified them as cancer patients.

I asked her [nurse] something, she was very brush off, you know, very abrupt. But I felt that when she treated White patients, it was different because you could see the mannerism. You can see the body language. I know some people, you know, think ‘Yeah, OK. How do you know?’ But I think you can tell it's like anything in life…you can tell the body language of people, we're not stupid.

(ID10; 59 years, Asian Indian; UG)

Finally, there were instances of perceived interpersonal racism/discrimination and microaggressions (intentional or not) within the healthcare setting. Some participants felt that their gender and ethnic background (e.g., being a ‘Black woman’) influenced how some HCPs (regardless of their ethnic background) behaved towards them. Others felt that racism is embedded in the system and some actions are normalised with little attention to the impact this can have on patients.

I really feel like with him [consultant] and the nurse that was involved, I believe that they felt they could pick on me, that I was a Black woman, and I was. So what?

(ID15; 52 years, Black Caribbean, SC)

#### 3.2.3 Barriers in Healthcare Services

We identified different barriers that participants encountered when they navigated the cancer services. These included access barriers (e.g., participants' multiple presentations in primary care before they were referred for secondary tests, or difficulties in accessing their breast cancer nurses) and lack of tailored services (e.g., wigs and prostheses largely not being suitable for minority ethnic women). One participant explained feeling alienated because, in her view, services catered to the White majority:

I feel like it was very much geared towards…White patients…So I didn't wear a wig…I felt like it was not very representative of my background, and it does make you feel a bit isolated in that sense…they don't cater for me or my hair or what would suit me…

(ID11; 41 years, Asian Pakistani, SC)

Moreover, the provision of support at different levels (e.g., financial, psychological, and emotional care) was perceived as essential to cope with breast cancer. However, this was not always offered or easily accessible. Although participants appreciated the support provided by major charity organisations, many shared their frustrations and disappointments because often these groups were not fully representative of minority ethnic women.

Lack of a diverse workforce at all levels was also perceived as a challenge that contributed both towards participants' negative experiences and had a systemic impact. This was exemplified by the discomfort or lack of relatedness when patients who do not see professionals who look like them. There were also concerns relating to influencing services design because minority ethnic professionals do not often hold senior decision‐making roles within healthcare services.

#### 3.2.4 Influence of Cultural Beliefs

For participants, family and their communities were both pillars to help them through the diagnosis and treatment, and a burden due to the impact of cultural beliefs and behaviours on them. A key theme discussed by participants was that of cancer being a taboo and a hidden condition in their communities. This cultural belief determined how patients dealt with their diagnosis and how it affected them psychologically.

I didn't want the ‘C word’ at home and I kept on saying to my sister… ‘I know it's something that…I'm gonna have to tell her [daughter] at some point’…but I just didn't want that at home…I just wanted to keep it …Quiet…for now, you know? And I did that right up until surgery.

(ID08; 49 years, Black Caribbean, SC)

#### 3.2.5 Black Feminist Thought, Intersectionality, and Breast Cancer

Minority ethnic women's lived experiences along the cancer pathway were analysed and interpreted through the lens of BFT and intersectionality. We observed that participants' breast cancer journeys are complex and present multiple and repeated opportunities to experience inequities in care and outcomes. These inequities were consistent between and within groups, regardless of sociodemographic factors. Differences by age were not evident, although it is worth noting that younger participants with children shared experiences of parenting anxiety about the future care of their children. They also positively acknowledged the role of educational settings in supporting children with mothers with breast cancer.

We observed that participants were in constant negotiation with the healthcare system and their social networks. A system perceived to be designed for the White majority created power asymmetries and placed minority ethnic women in disadvantaged positions. Minority stressors (e.g., microaggressions, prejudice, racism) negatively influenced participants' care experience. Societal expectations of resilience and cultural beliefs also had detrimental effect on minority ethnic women, adding burden to their management of the disease. Through BFT and intersectional lenses, these observations point to historical and contemporary systems of oppression that continue to shape policy and practice and perpetuate breast cancer inequities affecting minoritised groups.

**FIGURE 1:**
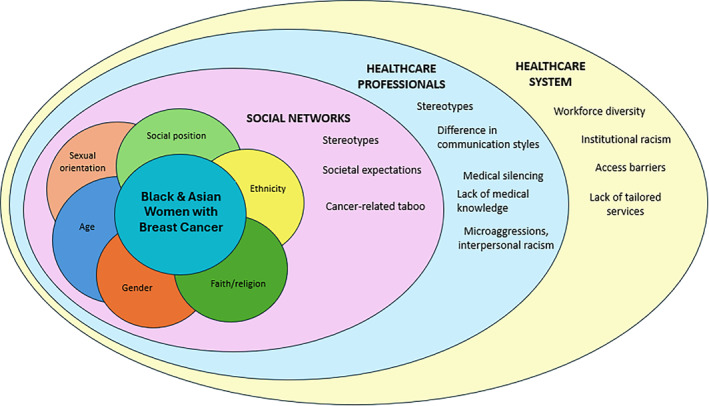
Visual representation of intersectional lived experiences of minority ethnic women with breast cancer in the UK.

Figure 1 provides a visual representation of the intersectional lived experience of minority ethnic women with breast cancer in England.

## 4 Discussion

There is an increasing interest on the role of multiple systems of discrimination to understand pathways to inequities in breast cancer care and outcomes [1, 7]. The present study identified how multifaceted factors combined in unique ways to influence the care experience and disease management for minority ethnic women in England. In addition, we provided a visual representation to illustrate how these multidimensional factors compounded to transform their experience of inequity along the breast cancer pathway (Figure 1).

Guided by the BFT and intersectionality, we explored the complex matrix of power within overlapping hierarchies of identity in relation to structural forces (healthcare system and social networks) that influenced minority ethnic women's breast cancer journeys. We observed an unequal distribution of power that intersects with historical and societal hierarchies to shape contemporaneous policies, services, and clinical practice [26, 27]. This complex matrix places Black and Asian women at increased opportunities of being disadvantaged and configures a system that perpetuates breast cancer inequities.

Furthermore, we identified a relevant equity gap within a publicly funded healthcare system that manifested through a lack of tailored services and inadequate provision of resources to patients from minority ethnic groups. These findings corroborate other evidence suggesting that often cancer health systems are not sufficiently responsive to the needs of minoritised women [28, 29]. Our study suggests that discrimination based on individual characteristics intersects with healthcare policies designed for the White majority and creates overlapping pathways into breast cancer inequities [7, 17]. Institutional discrimination and policy incongruence are systemic and widespread issues [17, 30]. Therefore, it is plausible that minority ethnic women with other cancers have similar experiences of inequity, and this warrants further investigation.

In line with existing evidence [31], participants shared how they dealt with multiple forms of stereotyping when they accessed cancer services. These instances occurred at multiple points along participants' journeys and accumulated over time. Stereotyping was linked to healthcare professionals' assumptions and racial‐gender‐social class bias [32]. Despite many participants expressing agency to deal with these experiences, this was an added burden and influenced their coping responses and breast cancer management. For instance, self‐silencing was a strategy to avoid being misunderstood. Self‐silencing is a coping strategy used by minority ethnic women to protect and preserve their wellbeing [33]. One main challenge of this strategy is that undermines women's agency to express their needs and engage in shared decision‐making.

Likewise, participants' accounts suggest there is embedded racism and discrimination in the healthcare system. These instances manifested in professionals' attitudes and behaviours towards participants, insufficient medical knowledge concerning breast cancer management in minority ethnic women, and participants' perception that the system is designed for the White majority. Consequently, many women felt alienated from the system [34], perceived they were medically silenced [25], and showed higher levels of psychological stress and poorer emotional well‐being [35]. These findings reinforce other evidence suggesting that institutional racism is a global issue [36], and intersectional experiences of alienation, undermined autonomy and competency, and minority stress are associated with inequities in breast cancer outcomes [31, 34].

Finally, the network system surrounding minority ethnic women played a key role in how participants dealt with and managed their disease. For instance, societal expectations of participants to be strong [37] were perceived as problematic and an added burden that they had to deal with alongside managing their breast cancer. This expectation was described as the ‘Strong Black Woman’ stereotype, which has historical roots, and has been evidenced to play a role in influencing the quality‐of‐life minority ethnic women with breast cancer [31]. Cancer considered as taboo and a hidden disease was another added burden to participants. For some participants this situation made them feel alienated as they could not seek advice and receive support from community members. Others were protective of their family and avoided naming and delayed sharing their cancer diagnosis. Therefore, we expand the existing literature around cancer‐related taboo and stigma [3] by underscoring the social dynamics minority ethnic women navigate through their breast cancer journeys.

### 4.1 Strengths and Limitations

Guided by the BFT and intersectionality, we provided a deeper and holistic understanding of the lived experiences of minority ethnic women along the breast cancer care pathway. Moreover, we contextualised individual experiences in relation to how structural forces have shaped contemporary healthcare services and lived experiences. Our collaboration with PPIE groups and two patient representatives who advised on the recruitment strategy and participant‐facing documents, and validated our interpretations of interview data findings and conclusions further strengthened the study.

Some limitations bear mentioning. We reported the experiences of minority ethnic women who participated in this study, and this may not be generalisable to all women from these communities. This study reported the challenges encountered by minority ethnic women living in England. Although these findings are country‐specific, they are consistent with other studies suggesting these challenges are possibly systemic and may be explained by sociohistorical events that continue to shape contemporary services and lived experiences for these groups [31, 34, 38].

### 4.2 Clinical Implications

Our findings suggest that unconscious bias within healthcare settings, explicit and implicit microaggressions and racist attitudes and behaviours, and cross‐cultural care challenges require attention. These issues can be addressed by integrating antiracism and cultural humility training into professional education programmes [39] and diversifying the medical curriculum to equip future professionals to provide culturally responsive and equity‐focused care [40]. To improve minority ethnic women's relatedness, communication, and interactions with breast cancer services is fundamental to diversify the workforce, particularly to ensure that there is ethnicity and gender congruence with healthcare professionals [34]. Policymakers and providers should address the policy and services gap that systematically disadvantage minority ethnic women. Steps to achieve this goal include becoming cognisant of the intersectional and oppressive institutional forces driving unequal experiences of care; and reformulating policies and services to ensure these reflect the diverse population they serve [16, 29].

## 5 Conclusion

Through this study, we identified some institutional deficiencies that systematically disadvantage minority ethnic women and processes that sustain the breast cancer care gap. Moreover, we demonstrated how minority ethnic groups can be further disadvantaged within public healthcare services. Based on our findings, we recommend policymakers and providers take action to ensure all women have the same opportunity to receive optimal and high‐quality breast cancer care. The challenges and recommendations presented in this study will be of interest to policymakers and clinicians in other high‐income countries and countries with similar services to that in England.

## Author Contributions

M.E.F.d.M. conceived the idea and was responsible for designing and developing the study methods, recruiting participants, conducting and analysing interview data, and drafting and editing the manuscript. K.L.W., E.R. and A.M. aided in developing the study methods, contributed meaningfully to the drafting and editing, and approved the final manuscript. S.G. and M.F. aided in designing the recruitment strategy and recruiting participants, interpreting and validating findings, contributed to the drafting and editing, and approved the final manuscript.

## Ethics Statement

This study was approved by the University of Surrey (Reference FHMS 22–23 018 EGA Amend 2) and the NHS Ethics Committees (Reference 23/YH/0153/AM01).

## Conflicts of Interest

The authors declare no conflicts of interest.

## Supporting information

Table S1

Table S2

## Data Availability

The authors have nothing to report.
