# Supplementary material for: Applying the Intersectionality Lens to Understand Minority Ethnic Women's Experiences of the Breast Cancer Care Pathway in England: A Qualitative Interview Study
Source: Psychooncology. 2025 Feb 6;34(2):e70092. doi: 10.1002/pon.70092 (PMC11803129; doi:10.1002/pon.70092)
Supplement: Supplementary file 1 — Table S1 [file PON-34-e70092-s002.docx]

Table Supplemental S1. Interview topic guide

| **Introduction and background questions to get to know participants** |
| --- |
| 1. Where do you live? |
| 1. Where did you grow up? Did you migrate to the UK? |
| 1. Do you work, study, and/or have care responsibilities? |
| 1. How do you describe yourself (self-identify)? |
| **Experience of breast cancer journey** |
| 1. How did you come to be diagnosed with breast cancer? |
| 1. Could you run me through the day you received your diagnosis? How did you feel? Did you share your diagnosis with your family/friends? Did you feel supported? |
| 1. Could you describe your medical team? How did it make you feel? |
| 1. Since you started with your treatment, how has it been for you to attend your appointments and manage your treatment? |
| 1. Could you explain to me what type of support information you have received? (e.g., financial support, wigs, prosthesis, support groups, psychological/emotional support) |
| 1. How has it been to live with breast cancer as a [Black/Asian/mixed ethnicity] woman? |
| **General questions** |
| 1. Do you think it is important for healthcare professionals to understand the role of faith or spirituality in patients going through breast cancer? Why? |
| 1. Do you perceive the experience with breast cancer services has been the same for all women? Could you explain to me why? In what ways? |
| 1. What advice would you give to other women about breast cancer and dealing with breast cancer services? |
| 1. Would you like to share any final highlights or experiences you had with NHS breast cancer patients? |
| 1. Have you completed the National Cancer Patient Experience Survey? |
| 1. Have you received a written cancer plan? |
