# Supplementary material for: Applying the Intersectionality Lens to Understand Minority Ethnic Women's Experiences of the Breast Cancer Care Pathway in England: A Qualitative Interview Study
Source: Psychooncology. 2025 Feb 6;34(2):e70092. doi: 10.1002/pon.70092 (PMC11803129; doi:10.1002/pon.70092)
Supplement: Supplementary file 2 — Table S2 [file PON-34-e70092-s001.docx]

**Supplemental Table S2.** [**Standards for Reporting Qualitative Research Checklist (SRQR)**](https://journals.lww.com/academicmedicine/fulltext/2014/09000/standards_for_reporting_qualitative_research__a.21.aspx)

| **No.** | **Topic** | **Page** |
| --- | --- | --- |
|  | **Title and abstract** |  |
| **S1** | Title | 1 |
| S2 | Abstract | 1 |
|  | **Introduction** |  |
| **S4** | Problem formulation | 2 |
| **S5** | Purpose of research question | 3 |
|  | **Methods** |  |
| **S5** | Qualitative approach and research paradigm | 2-3 |
| **S6** | Researcher characteristics and reflexivity | 4-5 |
| **S7** | Context | 1 |
| **S8** | Sampling strategy | 3-4 |
| **S9** | Ethical issues pertaining to human subjects | 15 |
| **S10** | Data collection methods | 3-4 |
| **S11** | Data collection instruments and technologies | 3-4 |
| **S12** | Units of study | 3-4 |
| **S13** | Data processing | 4 |
| **S14** | Data analysis | 4 |
| **S15** | Techniques to enhance trustworthiness | 4-5 |
|  | **Results/findings** |  |
| **S16** | Synthesis and interpretation | 5-11 |
| **S17** | Links to empirical data | 5-11 |
|  | **Discussion** |  |
| **S18** | Integration with prior work, implications, transferability, and contribution(s) to the field | 12-13 |
| **S19** | limitations | 13-14 |
|  | **Other** |  |
| **S20** | Conflicts of interest | 15 |
| **S21** | Funding | 15 |
